# Image denoising substantially improves accuracy and precision of intravoxel incoherent motion parameter estimates

**DOI:** 10.1371/journal.pone.0175106

**Published:** 2017-04-05

**Authors:** Carolin Reischauer, Andreas Gutzeit

**Affiliations:** 1 Institute of Radiology and Nuclear Medicine, Clinical Research Unit, Hirslanden Hospital St. Anna, Lucerne, Switzerland; 2 Institute for Biomedical Engineering, ETH and University of Zurich, Zurich, Switzerland; 3 Department of Radiology, Paracelsus Medical University Salzburg, Salzburg, Austria; Beijing University of Technology, CHINA

## Abstract

Applicability of intravoxel incoherent motion (IVIM) imaging in the clinical setting is hampered by the limited reliability in particular of the perfusion-related parameter estimates. To alleviate this problem, various advanced postprocessing methods have been introduced. However, the underlying algorithms are not readily available and generally suffer from an increased computational burden. Contrary, several computationally fast image denoising methods have recently been proposed which are accessible online and may improve reliability of IVIM parameter estimates. The objective of the present work is to investigate the impact of image denoising on accuracy and precision of IVIM parameter estimates using comprehensive in-silico and in-vivo experiments. Image denoising is performed with four different algorithms that work on magnitude data: two algorithms which are based on nonlocal means (NLM) filtering, one algorithm that relies on local principal component analysis (LPCA) of the diffusion-weighted images, and another algorithms that exploits joint rank and edge constraints (JREC). Accuracy and precision of IVIM parameter estimates is investigated in an in-silico brain phantom and an in-vivo ground truth as a function of the signal-to-noise ratio for spatially homogenous and inhomogenous levels of Rician noise. Moreover, precision is evaluated using bootstrap analysis of in-vivo measurements. In the experiments, IVIM parameters are computed a) by using a segmented fit method and b) by performing a biexponential fit of the entire attenuation curve based on nonlinear least squares estimates. Irrespective of the fit method, the results demonstrate that reliability of IVIM parameter estimates is substantially improved by image denoising. The experiments show that the LPCA and the JREC algorithms perform in a similar manner and outperform the NLM-related methods. Relative to noisy data, accuracy of the IVIM parameters in the in-silico phantom improves after image denoising by 76–79%, 79–81%, 84–99% and precision by 74–80%, 80–83%, 84–95% for the perfusion fraction, the diffusion coefficient, and the pseudodiffusion coefficient, respectively, when the segmented fit method is used. Beyond that, the simulations reveal that denoising performance is not impeded by spatially inhomogeneous levels of Rician noise in the image. Since all investigated algorithms are freely available and work on magnitude data they can be readily applied in the clinical setting which may foster transition of IVIM imaging into clinical practice.

## Introduction

Using diffusion-weighted imaging (DWI), the apparent diffusion coefficient (ADC) can be calculated which is a measure of tissue diffusivity and has been shown to be a viable biomarker for various pathological conditions. For instance, the ADC shows great promise for characterizing tumor masses and evaluating response to therapy at an early stage in head and neck tumors [1, 2] and brain cancers [3]. However, it has long been recognized that the ADC integrates the effects of diffusion and perfusion due to the pseudorandom organization of the capillary network at the voxel level [4, 5]. For this reason, Le Bihan et al. proposed the concept of intravoxel incoherent motion (IVIM) imaging. Signal attenuation due to diffusion weighting is thereby modelled as [4, 5]:

$$\text{S}(\text{b})={\text{S}}_{0}((1-\text{f})∙\text{exp}\left(-\text{b}∙\text{D}\right)+\text{f}∙\text{exp}\left(-\text{b}∙{\text{D}}^{*}\right)),$$ (1)

where S_0_ relates to the signal without diffusion weighting, f denotes the perfusion fraction, D is the diffusion coefficient, and D* corresponds to the pseudodiffusion coefficient. The first term describes signal decay due to diffusion in the intra- and extracellular tissue compartments and the second term relates to the so-called pseudodiffusion phenomenon. Due to the pseudodiffusion coefficient D* typically being an order of magnitude greater than the diffusion coefficient D, both compartments can be separated. Le Bihan and Turner established a link between the product of the perfusion fraction and the pseudodiffusion coefficient and the relative perfusion or blood flow [6]. In this manner, IVIM imaging permits separating the effects of diffusion and perfusion and may lead to a more comprehensive and differentiated understanding of the underlying tissue pathology and of alterations that occur in response to treatment.

However, clinical applicability of IVIM imaging is hampered by the limited reliability in particular of the perfusion-related parameter estimates if the biexponential fit is performed using iterative nonlinear least squares methods [7, 8]. One possibility to increase robustness of the results is to compute averaged values over regions of interest rather than individual voxels [7, 8]. Another approach that preserves spatial resolution is to use a segmented fit method as proposed by Pekar et al. [7]. It is likely the most frequently used algorithm in IVIM analysis. It relies on the fact that the decay rate resulting from pseudodiffusion is usually an order of magnitude greater than that stemming from tissue diffusion. For this reason, pseudodiffusion dominantly affects signal attenuation at lower b-values while it accounts for only a small proportion of the measured signal at higher b-values. Thus, the diffusion coefficient can be derived from a monoexponential fit of the high b-value images (typically > 200 s/mm^2^). Thereafter, the perfusion fraction is determined using the intercept obtained in the fit and the actual measurement without diffusion weighting. Finally, the pseudodiffusion coefficient is derived from a biexponential fit using the previously calculated values of the diffusion coefficient and the perfusion fraction.

Alternatively, it was shown that estimation uncertainty may be reduced relative to nonlinear least squares methods when a Bayesian probability approach is used for model fitting [9–12]. Furthermore, Freiman et al. have shown that combining a spatially-constrained incoherent motion model with an iterative fusion bootstrap solver results in more precise estimates of IVIM parameters [13]. However, the underlying algorithms are not readily available and suffer from an increased computational burden. Contrary, several computationally fast image denoising methods have recently been proposed which are accessible online (see Materials and Methods section) and may improve reliability of IVIM parameter estimates [14–19]. Up to now, image denoising as a means to increase accuracy and precision of IVIM modeling has not been evaluated and will be investigated in the current work.

Image denoising can be applied to either complex or magnitude diffusion-weighted (DW) images. Even though denoising of complex images allows for an easier modeling of the noise characteristics, it is preferable from a practical point of view to denoise magnitude images since they are widely available and require less storage. In many cases, noise in DW magnitude images follows a Rician distribution such as in images from a single coil [20–22] and in multiple-coil images reconstructed with sensitivity encoding (SENSE) [23]. Based on this assumption several algorithms have been proposed [24–26]. To improve denoising performance, a variety of constraints that exploit prior information can be incorporated. A well-known approach uses nonlocal similarities within the image [27]. The high computational burden that is associated with the method can be diminished by an optimized blockwise implementation of the algorithm [14]. The original version of this algorithm relied on the assumption of a Gaussian distribution of the noise in the image. To facilitate applicability to DW images, Wiest-Dasslé et al. proposed an adapted version which incorporates a Rician distribution of the noise [15]. In the following, this algorithm will be referred to as the nonlocal means (NLM) algorithm. The NLM algorithm assumes spatially homogenous levels of Rician noise. In practice, however, DWI acquisition is typically combined with parallel imaging to diminish susceptibility-induced image artifacts and T_2_* blurring [28, 29] which leads to varying levels of Rician noise across the image. For this reason, Manjón et al. proposed the adaptive nonlocal means (ANLM) algorithm [16]. As part of the denoising procedure, the ANLM algorithm inherently determines estimates of the noise variance on a voxelwise basis.

Alternative approaches exploit the multidirectional nature of DW images for image denoising. For instance, Manjón et al. demonstrated that magnitude DW images can be effectively denoised using local principal component analysis (LPCA) [17]. The LPCA algorithm assumes a Rician distribution of the noise in the image and accounts for spatially varying noise patterns. Beyond that, Lam et al. showed that the signal-to-noise ratio (SNR) of DW images can be effectively improved using joint rank and edge constraints (JREC) [18, 19]. Contrary to the aforementioned algorithms, the noisy magnitude images are modeled by a noncentral χ distribution of which the Rician distribution is a special case.

The aim of the present study is to evaluate the impact of image denoising on accuracy and precision of IVIM parameter estimates using comprehensive in-silico and in-vivo experiments. Image denoising is performed using the NLM, the ANLM, the JREC, and the LPCA algorithms. All investigated algorithms are freely available and work on magnitude data. Thus, they can be readily applied in the clinical setting which may foster transition of IVIM imaging into clinical practice.

## Materials and methods

Denoising performance of the NLM, the ANLM, the LPCA (available for download at: https://sites.google.com/site/pierrickcoupe/softwares/denoising-for-medical-imaging/mri-denoising/mri-denoising-software, accessed 23 May 2016), and the JREC (available for download at: http://mri.beckman.uiuc.edu/software.html, accessed 23 May 2016) algorithms was assessed with regard to accuracy and precision of the IVIM parameter estimates. IVIM parameters were computed twofold a) by using the segmented fit method and b) by performing a biexponential fit of the entire attenuation curve based on nonlinear least squares estimates. The latter will hereafter be referred to as a full biexponential fit.

### In-silico simulations

Denoising performance of the algorithms was investigated in a three-dimensional brain phantom (matrix = 144 x 144 x 10) based on the discrete version of the digital brain phantom created by Collins et al. (available for download at: http://brainweb.bic.mni.mcgill.ca/brainweb/anatomic_normal.html, accessed 23 May 2016) [30]. The IVIM parameters in the phantom were set to recent literature values **(Table 1)** [31]. Based on Eq 1, DW images of the brain phantom were computed with b-values of 0, 15, 30, 45, 60, 100, 250, 400, 550, 700, 850, 1000 s/mm^2^. Different levels of spatially homogeneous and inhomogeneous Rician noise were simulated by adding white Gaussian noise to the real and imaginary parts of the DW images resulting in a SNR range of = 20–100 in the non-DW images. Spatially varying noise distributions were generated similar to those described by Tabelow et al. [32]. In the simulations, the noise standard deviation was set relative to the joint average of the signals of gray and white matter in the non-DW images of the brain phantom. In clinical practice, DW images are usually acquired along three orthogonal directions to compensate for the possible influence of the relative orientation between tissue and imaging system [33]. For this reason, three noisy DW images were computed at each b-value and subsequently combined into a trace image.

**Table 1:** IVIM Parameters in the In-Silico Brain Phantom.

	f	D	D*
	[no units]	[x10^-3^ mm^2^/s]	[x10^-3^ mm^2^/s]
**Gray matter**	0.14	0.84	8.2
**White matter**	0.07	0.77	7.9

An estimate of the noise variance of each DW image series was determined using the median absolute deviation estimator adapted for Rician noise [34] and used as input parameter for the NLM and the JREC algorithms. The rank model order and the regularization parameter in the JREC algorithm were optimized at each SNR such that accuracy of the IVIM parameter estimates was maximized compared to the gold standard. The other image denoising algorithms were run with their default parameters.

Each noisy DW image series was denoised using all investigated algorithms and the IVIM parameters calculated. For comparison with previously published data, using the segmented fit method, the diffusion coefficients D were computed from the DW images with b > 250 s/mm^2^ [31]. In addition, IVIM parameters were computed from the noisy and noise-free DW image series, respectively. The latter were treated as gold standard for determining accuracy of parameter estimation. For each SNR, the simulation was repeated 50 times. Thereafter, accuracy and precision were computed jointly over gray and white matter.

The simulations were implemented and run in MATLAB (MathWorks, Natick, MA, USA, Release 2015a). To decrease computation time, the code was parallelized and distributed using MATLAB’s Parallel Computing Toolbox and Distributed Computing Server for Amazon EC2 (MathWorks, Natick, MA, USA, Release 2015a). Nonlinear least squares fitting for parameter estimation was implemented using the levmar package (Version 2.5) which comprises a C/C++ implementation of the Levenberg-Marquardt algorithm [35].

### In-vivo experiments

#### DWI measurements

For in-vivo comparison of the image denoising algorithms, DWI data of the brain of a healthy volunteer were acquired on a 3 T MR scanner (Achieva, Release 3.2.1, Philips Healthcare, Best, the Netherlands) using a 32-channel receive-only head coil array (Philips Healthcare, Best, the Netherlands). Data acquisition was approved by the Cantonal Research Ethics Committee and written informed consent was obtained from the volunteer. A standard DW spin-echo echo planar imaging (EPI) sequence with 12 b-values (b-values of 0, 15, 30, 45, 60, 100, 250, 400, 550, 700, 850, 1000 s/mm^2^) separately applied along three orthogonal directions was used. The scan parameters were as follows: field of view = 220 x 220 mm^2^, image matrix = 140 x 140, slices = 16, slice thickness = 5 mm, no slice gap, partial Fourier encoding = 75%, SENSE factor = 2, TR = 2000 ms, TE = 64 ms, number of signal averages (NSA) = 1, scan duration = 72 s. The scan was repeated 50 times to permit evaluating precision of IVIM parameter estimation using the bootstrap method [36]. Eddy current-induced image warping and motion were corrected and all data sets coregistered using a correlation-based affine registration algorithm [37]. The SNRs in a single measurement were determined according to the National Electrical Manufacturers Association [38]. Thereby, the SNR was calculated on a voxelwise basis using the ratio of the mean signal intensity of a given voxel over all scan repetitions divided by the standard deviation around that mean.

#### Bootstrap analysis of in-vivo data

The non-DW images were averaged and calculated for the probability of belonging to either gray matter, white matter or cerebrospinal fluid on a voxelwise basis using SPM (Statistical Parametric Mapping, SPM8, Wellcome Department of Cognitive Neurology, London, UK). The white matter probability mask was smoothed using a three-dimensional Gaussian kernel with a full width at half maximum of 3 mm to mitigate partial volume effects [31, 39]. A joint gray and white matter mask was thereafter created at a threshold of 0.9.

Bootstrapping was used to assess in-vivo precision of IVIM parameter estimates as a function of the NSA (NSA = 2–25). For each NSA, the corresponding number of scan repetitions was randomly selected out of the 50 repetitions and averaged to create a bootstrap estimate of the DW image series. The procedure was repeated to generate a total of 50 bootstrap estimates with replacement at each NSA. It should be noted that scan repetitions rather than individual b-values were randomly picked and averaged to account for signal drifts as a results of temporal scanner instability [40]. Each bootstrap estimate was denoised using all evaluated algorithms and the IVIM parameters calculated. In addition, IVIM parameter estimates were also derived directly from the noisy bootstrap estimates of the DW image series. In the analysis, the rank model order in the JREC algorithm was set to 4 and the regularization parameter was chosen by visual inspection of the IVIM parameter maps to avoid oversmoothing [18, 19].

#### Surrogate in-vivo ground truth

Due to the absence of a gold standard, accuracy of IVIM estimation cannot be determined in vivo. For comparison with the in-silico simulations, a ground truth was generated from the in-vivo data using a similar approach as proposed by Zhou et al. [41]. In the process, a) the DW image series were eddy-current corrected, motion compensated and coregistered, b) the DW image series were denoised using the JREC algorithm and subsequently averaged, and c) the IVIM parameter maps were calculated. The JREC algorithm was chosen for post processing since it allows a tradeoff between denoising performance and image smoothing. For computation of this ground truth, a relatively large regularization parameter was chosen to maximize noise removal while accepting some loss of image detail. Based on the thus generated in-vivo ground truth, the same simulations as in the in-silico phantom were run, i.e. the same b-values were assumed and different levels of spatially homogeneous and inhomogeneous Rician noise were added resulting in a SNR range of = 20–100 in the non-DW images. It should be noted that smaller regularization parameters were used when the JREC algorithm was used for image denoising in the simulations than when the ground truth was created.

### Measures of algorithm performance

In addition to a visual comparison of noisy and denoised IVIM parameter maps, two error metrics were computed to assess accuracy and precision of IVIM parameter estimation. To quantify parameter estimation accuracy, the root-mean-square error (RMSE) of the IVIM parameter estimates was computed:

$$\text{RMSE}=\sqrt{\frac{\sum_{\text{m}=1}^{\text{M}}{\left({\text{p}}_{\text{m}}\text{-}{\hat{\text{p}}}_{\text{m}}\right)}^{2}}{\text{M}}},$$ (2)

where p_m_ and ${\hat{\text{p}}}_{\text{m}}$ correspond to the IVIM parameter estimates in voxel m computed from the gold standard and the noisy/denoised DW images, respectively. M corresponds to the total number of gray and white matter voxels in the data set. For each SNR/NSA, the RMSE was calculated jointly over gray and white matter and subsequently averaged across all simulations (n = 50).

At each SNR/NSA, precision was assessed by computation of the coefficient of variation (CV):

$$\text{CV}=100\%∙\frac{\sum_{\text{m}=1}^{\text{M}}\sigma_{\text{m}}/\mu_{\text{m}}}{\text{M}},$$ (3)

where μ_m_ denotes the average value and σ_m_ the corresponding standard deviation of the IVIM parameter estimate in voxel m across all simulations (n = 50) and M relates to the total number of gray and white matter voxels in the data set.

## Results

### Visual comparison of IVIM parameter maps

**Fig 1:**
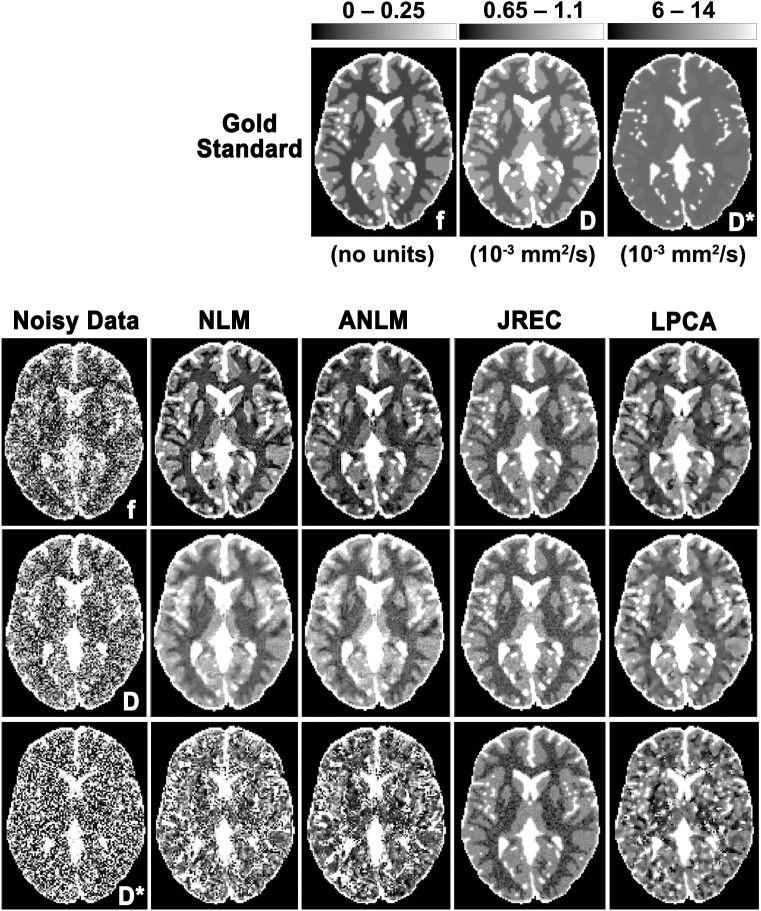
Parameter maps of the perfusion fraction f (first row), the diffusion coefficient D (second row), and the pseudodiffusion coefficient D* (third row) in the in-silico brain phantom computed from noisy (first column) and denoised DW images using the NLM (second column), the ANLM (third column), the JREC (fourth column), and the LPCA algorithms (fifth column). Stationary homogeneous Rician noise was added to obtain a SNR of 40 and the IVIM parameters were computed using the segmented fit method. The gold standard is depicted for reference.

For visual comparison, **Fig 1** depicts IVIM parameter maps computed from noisy and denoised DW image series when the segmented fit method is used. Quality of the IVIM parameter maps notably improves after image denoising. With regard to the pseudodiffusion coefficient, quality of the parameter map is highest when the JREC algorithm is utilized. However, it should be noted that relative to the gold standard the pseudodiffusion coefficient appears to be systematically underestimated in brain white matter **(Fig 1, third row)**.

**Fig 2:**
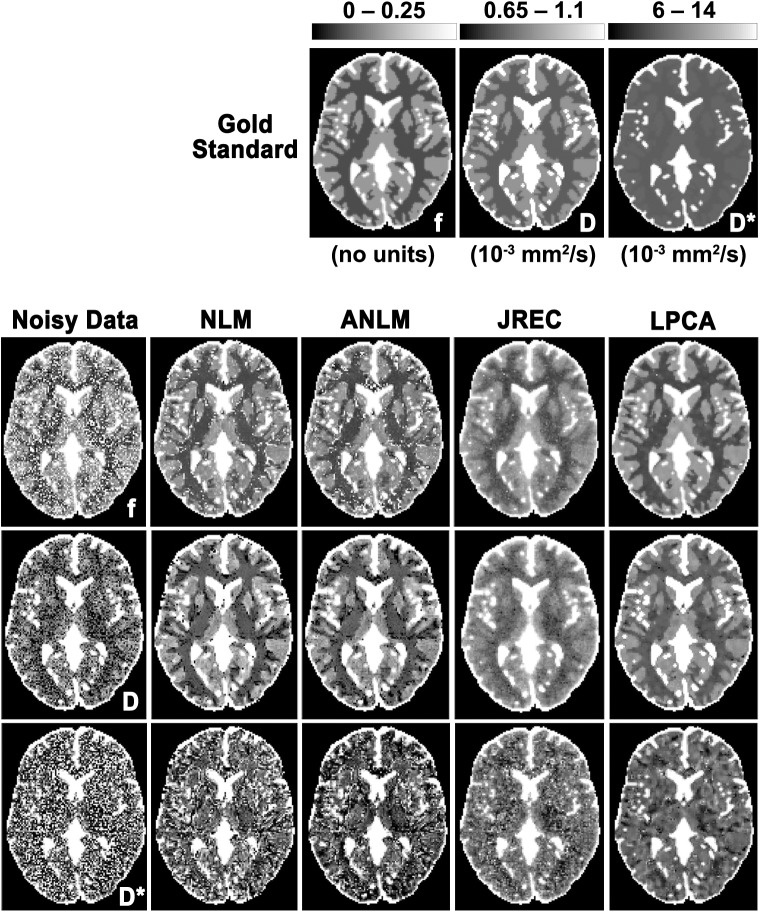
Parameter maps of the perfusion fraction f (first row), the diffusion coefficient D (second row), and the pseudodiffusion coefficient D* (third row) in the in-silico brain phantom computed from noisy (first column) and denoised DW images using the NLM (second column), the ANLM (third column), the JREC (fourth column), and the LPCA algorithms (fifth column). Stationary homogeneous Rician noise was added to obtain a SNR of 60 and the IVIM parameters were computed using a full biexponential fit. The gold standard is depicted for reference.

By way of example, **Fig 2** shows IVIM parameter maps computed from noisy and denoised DW image series when a full biexponential fit is performed. Visual inspection reveals that the highest image quality is achieved when using the LPCA algorithm for image denoising. However, even then image quality of the pseudodiffusion coefficient is relatively poor. Furthermore, it can be observed that both NLM-related algorithms lead to errors in the parameter estimates at transitions between different types of tissue.

### Accuracy of IVIM parameters after image denoising

#### In-silico brain phantom simulations

**Fig 3:**
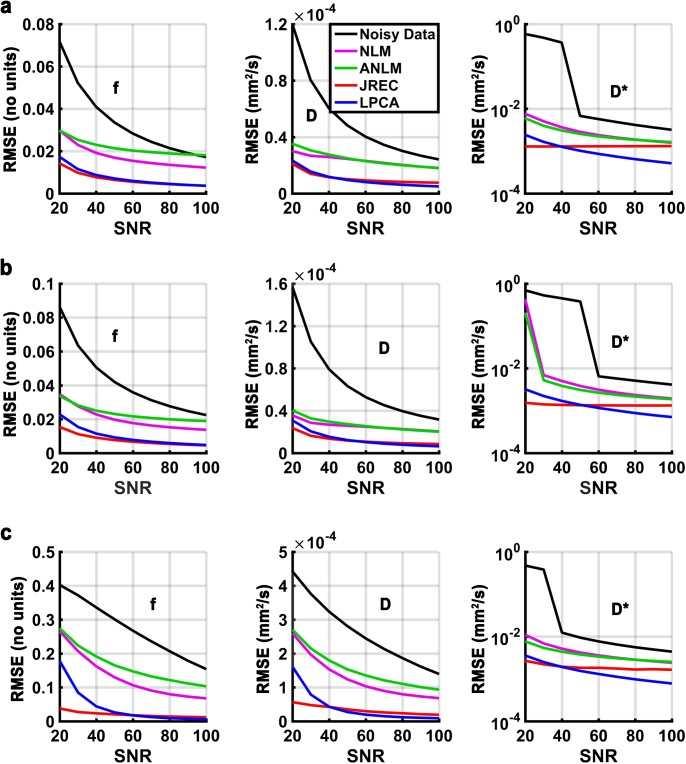
RMSEs of the perfusion fraction f (first column), the diffusion coefficient D (second column), and the pseudodiffusion coefficient D* (third column) in the in-silico brain phantom as a function of the SNR. Results of the simulations when (a) stationary and (b) spatially varying Rician noise is added and the segmented fit method is utilized, and (c) when stationary Rician noise is added and a full biexponential fit is performed. Please note that the RMSEs of the pseudodiffusion coefficient (third column) are displayed on a logarithmic scale for clarity. At each SNR, the RMSEs were calculated as the average across all simulations (n = 50).

**Fig 3** depicts the RMSEs of the IVIM parameter estimates in the in-silico brain phantom as a function of the SNR. It can be appreciated that independent of the fit method, accuracy of IVIM parameter estimation considerably improves after image denoising, in particular at low and moderate SNRs. The JREC and the LPCA algorithms result in similar RMSEs and generally perform better than the NLM-related methods. Relative to the noisy DW image series, accuracy improves by 76–79%, 79–81%, and 84–99% for the perfusion fraction f, the diffusion coefficient D, and the pseudodiffusion coefficient D*, respectively, across the simulated SNR range, if the DW image series is denoised with the LPCA algorithm prior to IVIM parameter estimationusing the segmented fit method **(Fig 3A)**. Thereby, denoising performance is not impeded by spatially varying levels of Rician noise **(Fig 3B)**. Despite image denoising, accuracy of IVIM parameter estimation based on a full biexponential fit is much lower than when the segmented fit method is used. Both fit methods achieve comparable results only at high SNRs **(Fig 3A and 3C)**. When the JREC algorithm is used for image denoising it should be noted with regard to the pseudodiffusion coefficient that a constant RMSE is observed that persists at high SNRs **(Fig 3, third row)**. This finding is a consequence of the systematic error in the parameter estimates in brain white matter, as discussed further up. For the pseudodiffusion coefficient D*, a critical SNR can be observed below which the RMSE increases strongly **(Fig 3, third row)**, this is likely caused by the Levenberg-Marquardt algorithm converging towards a local instead of the global minimum.

#### In-vivo ground truth simulations

Due to the lack of a gold standard, accuracy of IVIM parameter estimation cannot be determined in vivo. As noted above, a surrogate in-vivo ground truth was generated from the in-vivo measurements to assess transferability of the results of the in-silico simulations to the in-vivo situation. **Table 2** summarizes the IVIM parameters in the in-vivo ground.

**Table 2:** IVIM Parameters in the In-Vivo Ground Truth.

	f	D	D*
	[no units]	[x10^-3^ mm^2^/s]	[x10^-3^ mm^2^/s]
**Gray matter**	0.11 ± 0.06	0.76 ± 0.20	7.7 ± 4.2
**White matter**	0.07 ± 0.03	0.72 ± 0.20	7.2 ± 4.5

Please note, values in the in-vivo ground truth are expressed as mean ± standard deviation.

**Fig 4:**
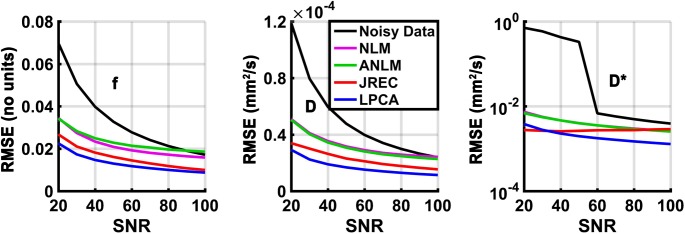
RMSEs of the perfusion fraction f (first column), the diffusion coefficient D (second column), and the pseudodiffusion coefficient D* (third column) in the in-vivo ground truth as a function of the SNR when spatially homogeneous Rician noise is added and a segmented fit is performed. Please note that the RMSEs of the pseudodiffusion coefficient (third column) are displayed on a logarithmic scale for clarity. At each SNR, the RMSEs were calculated as the average across all simulations (n = 50).

With regard to accuracy, the results of the in-silico experiments are corroborated in the in-vivo ground truth. By way of example, **Fig 4** depicts the RMSEs of the IVIM parameter estimates in the in-vivo ground truth when spatially homogeneous Rician noise is added and the segmented fit method is utilized. The results agree well with those of the in-silico brain phantom **(Figs 3A and 4)**. However, it should be noted that the RMSEs in the in-vivo ground truth were generally somewhat higher than in the in-silico brain phantom.

### Precision of IVIM parameters after image denoising

#### In-silico brain phantom simulations

**Fig 5:**
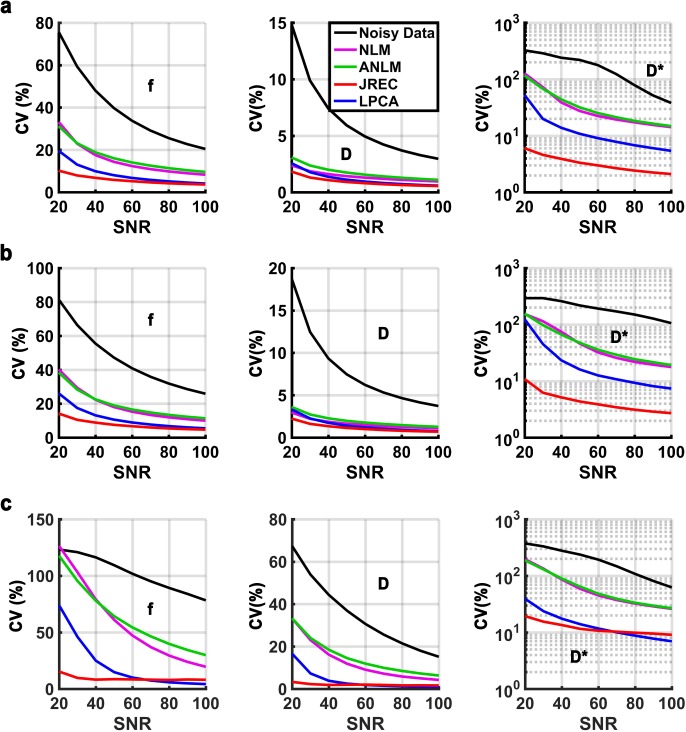
CVs of the perfusion fraction f (first column), the diffusion coefficient D (second column), and the pseudodiffusion coefficient D* (third column) in the in-silico brain phantom as a function of the SNR. Results of the simulations when (a) stationary and (b) spatially varying Rician noise is added and the segmented fit method is utilized, and (c) when stationary Rician noise is added and a full biexponential fit is performed. Please note that the CVs of the pseudodiffusion coefficient (third column) are displayed on a logarithmic scale for clarity.

**Fig 5** depicts the CVs of the IVIM parameter estimates in the in-silico brain phantom as a function of the SNR. As can be seen, image denoising considerably improves precision of the estimates. In agreement with the observations made with regard to accuracy, the JREC and the LPCA algorithms outperform the NLM-related methods. Relative to the noisy DW image series, precision is improved by 74–80%, 80–83%, and 84–95% for the perfusion fraction f, the diffusion coefficient D, and the pseudodiffusion coefficient D*, respectively, across the simulated SNR range, if the DW image series is denoised with the LPCA algorithm prior to IVIM parameter estimation using the segmented fit method **(Fig 5A)**. Furthermore, denoising performance is not impeded when spatially varying levels of Rician noise are added **(Fig 5B)**. Similar to before, precision of IVIM parameter estimation based on a full biexponential fit is much lower than when the segmented fit method is applied. Both fit methods achieve comparable results only at high SNRs **(Fig 5A and 5C)**.

#### In-vivo ground truth simulations

**Fig 6:**
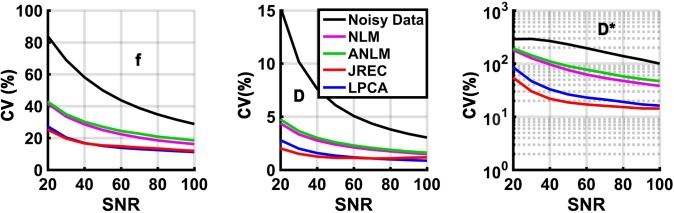
CVs of the perfusion fraction f (first column), the diffusion coefficient D (second column), and the pseudodiffusion coefficient D* (third column) in the in-vivo ground truth as a function of the SNR when spatially homogeneous Rician noise is added and a segmented fit is performed. Please note that the CVs of the pseudodiffusion coefficient (third column) are displayed on a logarithmic scale for clarity.

With regard to precision, the results of the in-silico experiments are corroborated in the in-vivo ground truth. By way of example, **Fig 6** depicts the CVs of the IVIM parameter estimates in the in-vivo ground truth when spatially homogeneous Rician noise is added and the segmented fit method is utilized. The results are in agreement with those of the in-silico brain phantom **(Figs 5A and 6)**. However, it should be noted that precision of the IVIM parameter estimates after image denoising with the JREC algorithm was generally higher in the in-silico brain phantom than in the in-vivo ground truth **(Figs 5A and 6)**.

#### Bootstrap analysis of the in-vivo measurements

**Fig 7:**
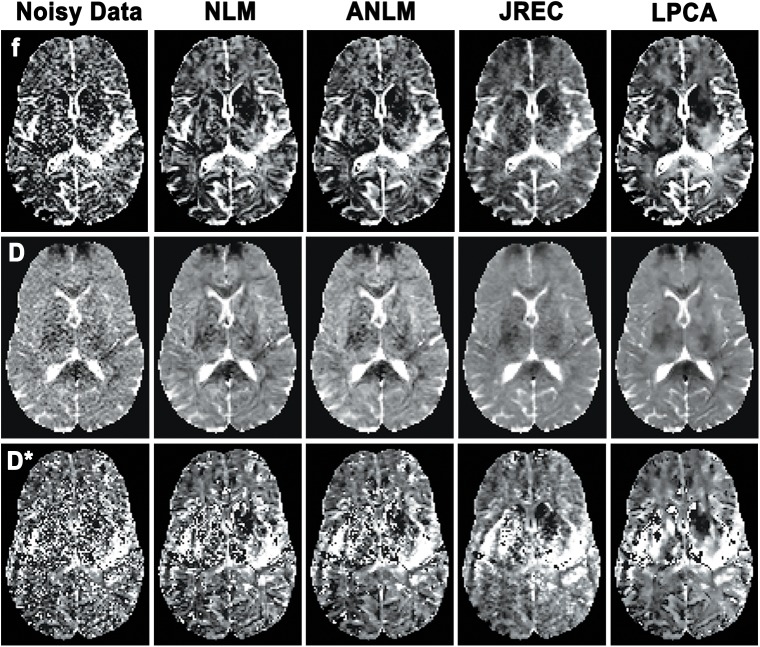
Parameter maps of the perfusion fraction f (first row), the diffusion coefficient D (second row), and the pseudodiffusion coefficient D∗ (third row) computed from noisy (first column) and denoised in-vivo DW images using the NLM (second column), the ANLM (third column), the JREC (fourth column), and the LPCA algorithms (fifth column). The results were computed from a single measurement (NSA = 1, scan duration = 72 s).

For visual comparison, Fig 7 depicts IVIM parameter maps computed from noisy and denoised in-vivo DW images when the segmented fit method is applied. The parameter maps were computed from a single measurement (NSA = 1, scan duration = 72s). Quality of the parameters maps clearly improves after image denoising. As before, visual inspection reveals that the JREC and the LPCA algorithms perform better that the NLM-related methods. The artifact that is apparent on the pseudodiffusion coefficient maps corresponds to a residual aliasing artifact after SENSE reconstruction likely caused by inaccurate estimation of the coil sensitivity maps.

The SNR computed jointly over gray and white matter amounted on average to 22.92 ± 4.12 in the non-DW images (NSA = 1). Please note that when neglecting diffusion, the SNR in the trace DW images is increased by a factor of square root of three relative to the non-DW images since diffusion weighting was sequentially applied along three orthogonal directions. Taking into consideration that the SNR scales with the square root of the NSA, the NSA range (NSA = 2–25) in the bootstrap analysis of the in-vivo measurements translates into a SNR range that agrees well with the in-silico simulations. By way of example, **S1 Fig** of the Supporting Information shows in-vivo SNR maps and **S1 Table** summarizes the results of the in-vivo SNR measurements.

**Fig 8:**
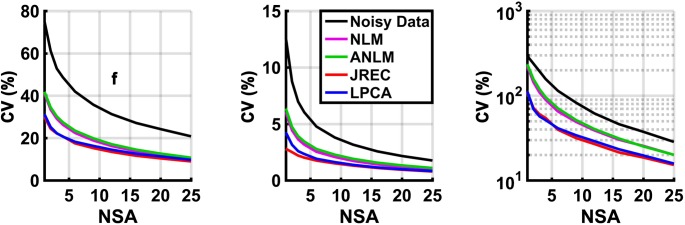
Results of the bootstrap analysis of the in-vivo data: CVs of the perfusion fraction f (first column), the diffusion coefficient D (second column), and the pseudodiffusion coefficient D∗ (third column) as a function of the NSA when IVIM parameter estimation is performed using the segmented fit method. Please note that the CVs of the pseudodiffusion coefficient (third column) are displayed on a logarithmic scale for clarity.

Finally, **Fig 8** depicts the results of the bootstrap analysis. In-vivo precision of the IVIM parameter estimates is slightly lower but generally agrees well with the in-silico simulations **(Figs 5A, 5B and 8).** Precision of the perfusion fraction f considerably increases after image denoising with either the LPCA or the JREC algorithm (CVs ≤ 20% for NSA ≥ 5). Moreover, it should be stressed that the CVs of the diffusion coefficient D were smaller than 5% across the entire NSA range after image denoising using the aforementioned algorithms.

## Discussion

In the present study, the impact of image denoising on the reliability of IVIM parameter estimates was investigated using comprehensive in-silico and in-vivo experiments. IVIM parameters were computed by using either the segmented fit method or by performing a biexponential fit of the entire attenuation curve. Irrespective of the fit method, the results demonstrate that both accuracy and precision are substantially improved after image denoising. It was shown that the LPCA and the JREC algorithms perform in a similar manner and outperform the NLM-related methods. Despite image denoising, reliability of IVIM parameter estimates is generally markedly lower when a full biexponential rather than a segmented fit is utilized. Both methods achieve similar results only at high SNRs which are unlikely to be obtained in most clinical applications.

In the present study, precision of IVIM parameter estimates was evaluated in an in-silico brain phantom. The results of these simulations were verified by bootstrap analysis of in-vivo measurements. Due to the lack of a gold standard, accuracy of the IVIM parameter estimates could not be determined in vivo. To investigate transferability of the results of the in-silico simulations to the in-vivo situation, a ground truth was generated from the in-vivo measurements in a similar manner as proposed by Zhou et al. [41]. The IVIM parameters in the resulting in-vivo ground truth agreed well with literature values (see Tables 1 and 2) [31, 42]. The simulations that were run in the thus generated in-vivo ground truth corroborated the findings in the in-silico experiments. Finally, the results of the present work with regard to accuracy of IVIM parameter derived from noisy data agree well with recently published data [31].

In the simulations, accuracy was determined relative to the IVIM parameters computed from the noise-free images. It should be noted that there is a systematic error relative to the gold standard when the segmented fit method is used. This inaccuracy stems from a residual amount of signal due to perfusion even at higher b-values and is inherent to the method [7].

Performance of the image denoising algorithms relies on an accurate estimate of the noise variance. The NLM and the JREC algorithms require this estimate as an input parameter. For this purpose, an object-based method was utilized in the present work. While background-based noise estimation methods are easily applicable they rely on reasonably large background regions which are representative of the noise level in the object. This assumption might not be met in DWI since image acquisition is typically performed using EPI which is prone to ghosting artifacts due to the misalignment of even and odd echoes. These artifacts may lead to erroneous results if background-based noise estimation methods are used.

As noted above, DWI is usually performed in conjunction with parallel imaging to diminish susceptibility-induced image artifacts and T_2_* blurring [28, 29] which leads to spatially varying levels of Rician noise in the image. Among the algorithms studied, only the ANLM and the LPCA algorithms permit estimating the noise variance on a voxelwise basis. However, the JREC algorithm could be readily adapted to allow for spatially varying levels of noise by introducing a voxel-dependent noise variance in combination with a noise estimation method that allows for a voxelwise determination of the noise level [18]. However, the present results indicate that denoising performance is not diminished by spatially inhomogeneous levels of Rician noise.

In many cases, noise in magnitude MR images follows a Rician distribution such as in images from a single coil [20–22] and in multiple-coil images reconstructed with SENSE [23]. However, for instance generalized autocalibrating partially parallel acquisition reconstruction with sum-of-squares combination follows a noncentral χ distribution. In this case, images can either be directly denoised using the JREC algorithm or image denoising may be performed prior to coil combination using the other evaluated denoising methods.

Reliability of the IVIM parameter estimates was evaluated assuming a DWI sequence with 12 b-values as described in Wu et al. [31]. While accuracy and precision may be increased by acquiring more b-values, the maximum number of b-values is in practice limited by time constraints in the clinical setting. Nevertheless, it should be noted that the simulations lead to comparable results when a large number of b-values (number of b-values = 25, data not shown) was assumed. For this reason, it is recommended to use image denoising as a postprocessing step independent of the number of acquired b-values.

The present work demonstrates that image denoising based on either the LPCA or the JREC algorithm results in more robust IVIM parameter estimates than when the NLM-related methods are used. Both algorithms make use of the multidirectional nature of DW images. Thus, misregistration among DW images with different b-values will lead to erroneous results. For this reason, it is important to correct in-vivo data for eddy current-induced image distortions and motion. To this end, a correlation-based affine registration algorithm was utilized in the present work [37].

In the in-vivo experiment, the parameters of the JREC algorithm were chosen by visual inspection of the IVIM parameter maps to avoid oversmoothing. In situations where a DW data set features particularly low SNRs, a higher regularization parameter may be chosen to increase noise removal at the expense of image detail. The NLM-related algorithms were run with their default parameters which were optimized in a previous work [14]. To increase denoising performance of the NLM-related methods Coupé et al. proposed a multiresolution framework with wavelet subband mixing [42]. However, preliminary simulations (data not shown) indicated only minor improvements in denoising performance at the cost of double the computational burden. For this reason, image denoising was performed without subband mixing in the present work.

Finally, it should be noted that image denoising could in principle be easily combined with parameter estimation using a Bayesian probability approach for model fitting. Previous work has shown that in this manner reliability of the IVIM parameters estimates may potentially be increased [9–12]. However, IVIM parameter estimation was in the present work performed by nonlinear least-squares fitting due to its computational efficiency and its widespread use.

In conclusion, the present work evaluated the impact of image denoising on the reliability of IVIM parameter estimates. Irrespective of the fit method, it was shown that both accuracy and precision are substantially improved when DW images are denoised prior to IVIM parameter fitting. It was observed that the LPCA and the JREC algorithms in conjunction with the segmented fit method result in the highest robustness of the IVIM parameter estimates. Both algorithms work on magnitude data and can thus be readily applied in the clinical setting which may foster transition of IVIM imaging into clinical practice.

## Supporting information

S1 Fig In-vivo SNR maps of three slices in the brain at b-values of 0, 100, 550, 1000 s/mm^2^. Please note that diffusion weighting at each b-value was applied along three orthogonal directions and the images were subsequently combined into a trace image.
(DOCX)

S1 Table Mean values and standard deviations of the SNRs in the in-vivo measurements jointly computed across gray and white matter (NSA = 1). (DOCX)

S2 Fig Flow chart depicting the simulation pipeline used in the present work. In addition, links to the locations of the source code that is used in the simulations are provided.
(PDF)
