# Supplementary material for: Image denoising substantially improves accuracy and precision of intravoxel incoherent motion parameter estimates
Source: PLoS One. 2017 Apr 5;12(4):e0175106. doi: 10.1371/journal.pone.0175106 (PMC5381911; doi:10.1371/journal.pone.0175106)
Supplement: S1 Fig — Please note that diffusion weighting at each b-value was applied along three orthogonal directions and the images were subsequently combined into a trace image. (DOCX) [file pone.0175106.s001.docx]

**
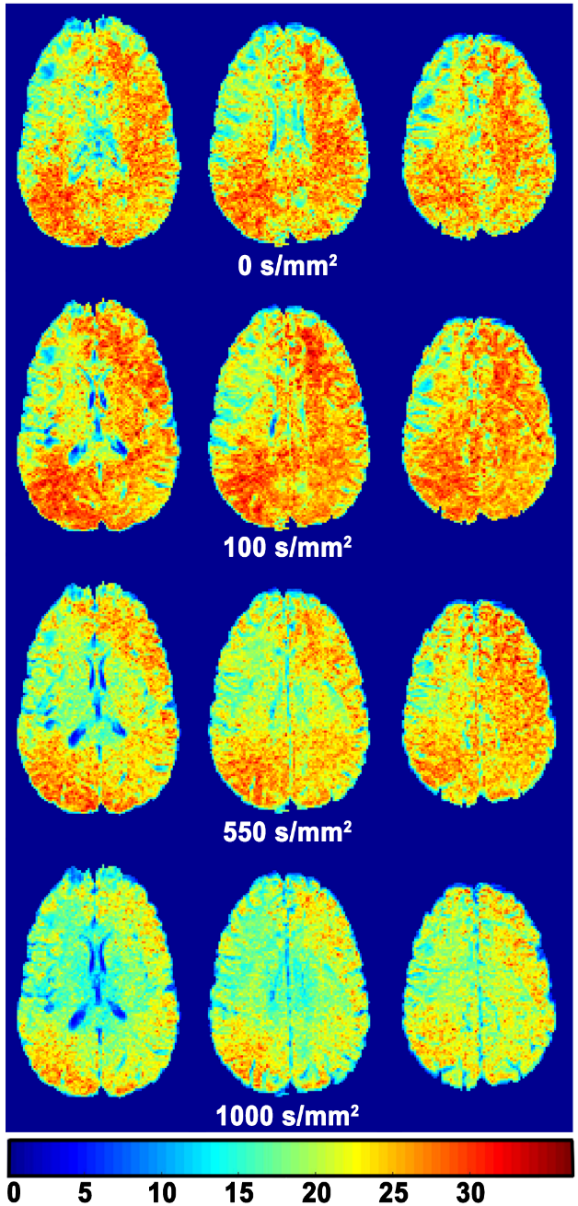
**

**S1 Fig**

In-vivo SNR maps of three slices in the brain at b-values of 0, 100, 550, 1000 s/mm^2^. Please note that diffusion weighting at each b-value was applied along three orthogonal directions and the images were subsequently combined into a trace image.
