# Supplementary material for: Image denoising substantially improves accuracy and precision of intravoxel incoherent motion parameter estimates
Source: PLoS One. 2017 Apr 5;12(4):e0175106. doi: 10.1371/journal.pone.0175106 (PMC5381911; doi:10.1371/journal.pone.0175106)
Supplement: S1 Table — (DOCX) [file pone.0175106.s002.docx]

**S1 Table**

Mean values and standard deviations of the SNRs in the in-vivo measurements jointly computed across gray and white matter (NSA = 1).

| **b-value** | **Mean value** | **Standard deviation** |
| --- | --- | --- |
| **[s/mm^2^]** | **of the SNR** | **of the SNR** |
| 0 | 22.92 | 4.12 |
| 100 | 24.31 | 4.01 |
| 550 | 21.83 | 3.80 |
| 1000 | 19.14 | 3.58 |
|  | | |
