# Supplementary material for: Image denoising substantially improves accuracy and precision of intravoxel incoherent motion parameter estimates
Source: PLoS One. 2017 Apr 5;12(4):e0175106. doi: 10.1371/journal.pone.0175106 (PMC5381911; doi:10.1371/journal.pone.0175106)
Supplement: S2 Fig — In addition, links to the locations of the source code that is used in the simulations are provided. (PDF) [file pone.0175106.s003.pdf]

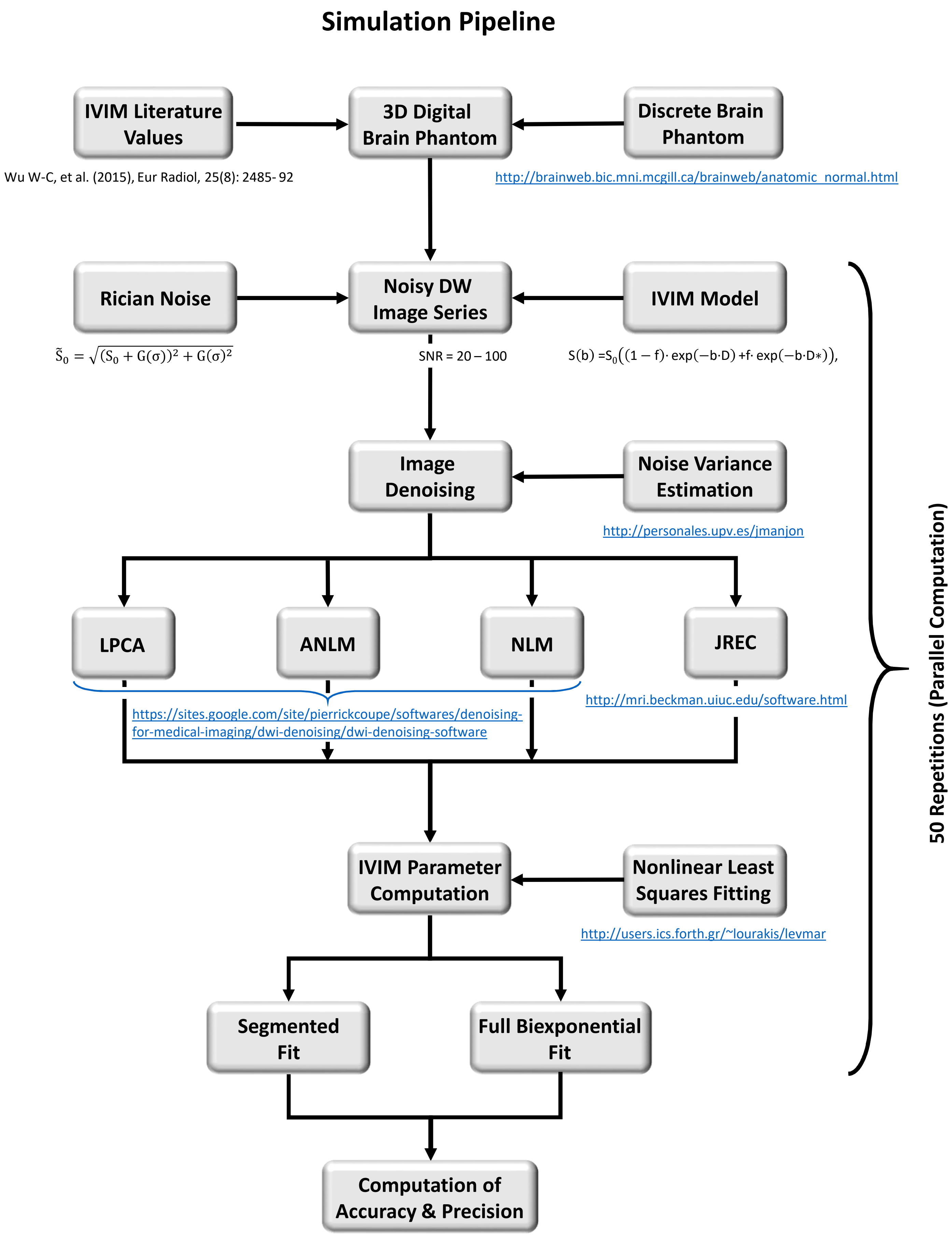

S2 Fig

Flow chart depicting the simulation pipeline used in the present work. In addition, links to the locations of the source code that is used in the simulations are provided.
